# Lessons learned from developing serious games for upper-limb rehabilitation in children

**DOI:** 10.3389/fped.2026.1904423

**Published:** 2026-10-21

**Authors:** David Infante Sanchez, Aikaterini Bourazeri

**Affiliations:** 1 Electronic Engineering, Technological Institute of Morelia, Morelia, Mexico; 2 School of Computer Science and Electronic Engineering, University of Essex, Wivenhoe Park, United Kingdom

**Keywords:** game design considerations, human-centered design, rehabilitation in children, serious games, therapeutic gamification

## Abstract

**Background:**

Virtual reality (VR), augmented reality (AR), and serious games have demonstrated growing potential in physical therapy, exergaming, and the promotion of physical activity across diverse patient populations. However, the development of therapeutic games, particularly for rehabilitation in children, still lacks consolidated guidelines and structured frameworks.

**Objective:**

This study aims to document the design and development process of two gamified upper-limb rehabilitation systems for children, developed in collaboration with physical therapists, and to derive preliminary clinician-informed design recommendations.

**Methods:**

An iterative development process was carried out in close collaboration with clinicians. Feedback and observations from therapy sessions were collected and analyzed. These findings were contextualized through a targeted narrative review of relevant literature to examine their relationship with existing research.

**Results:**

The comparative analysis produced actionable design recommendations regarding game mechanics, reward structures, adaptive difficulty, and feature extraction strategies. Key findings highlight the need to tailor gameplay to children's abilities through simplified narratives, therapeutic mechanics, consistent feedback, and adaptive difficulty.

**Conclusions:**

The results highlight the unique challenges and opportunities involved in designing serious games for therapy for children. It advocates for the development of preliminary clinician-informed design principles to support the creation of engaging, usable, and clinically grounded rehabilitation games for children.

## 1 Introduction

Serious games have played an increasingly important role in physical rehabilitation by supporting patient engagement and enabling the measurement of physical performance through electronic and optical sensing technologies. Motion-capture systems using inertial measurement units (IMUs) and optical cameras, combined with computer games, have been applied to upper- and lower-limb rehabilitation across conditions including sports injuries, gait impairments, stroke, multiple sclerosis, and spasticity (1). Game-based technologies have also been used with children; for example, handheld devices such as the Wii Remote combined with Nintendo games have been employed to support balance rehabilitation in children with cerebral palsy (2). Such approaches may be particularly valuable in rehabilitation involving children, where motivation, age-appropriate interaction, and sustained engagement are important considerations.

Despite the growing use of these technologies, the development of serious games for rehabilitation lacks a specific and standardized framework, particularly for translating clinical and user requirements into practical design decisions. Game design involves interconnected elements including mechanics, dynamics, aesthetics, and emotional engagement, which influence motivation and goal-oriented behaviour (3, 4). Within rehabilitation, feedback is particularly important because it allows users to understand their progress through visual, auditory, or haptic cues and can support motivation and meaningful interaction (5). Clear visual and auditory rewards may also be beneficial for users with learning disabilities (6). Previous research has therefore emphasized simple gameplay and narratives (7), multimodal feedback, positive reinforcement, and close correspondence between therapeutic movements and in-game actions (8–10).

Rehabilitation games must also accommodate substantial differences in users’ physical and cognitive abilities. Relevant design principles include integrating therapeutic gestures into the virtual environment, calibrating game mechanics to users’ motor capabilities, monitoring and recording performance, progressively adapting challenge, providing appropriate cognitive and sensory feedback, and using positive rewards to support engagement (11). Customizable settings for movement range, speed, and recovery time are particularly relevant in rehabilitation contexts (12), while limited customization and excessive visual stimulation can reduce the suitability of rehabilitation technologies for users with specific functional needs (13). These considerations reinforce the importance of designing systems around the characteristics of the intended users, including their abilities, preferences, knowledge, and motivation (14).

Difficulty and pacing represent additional challenges. Tasks that are too difficult may contribute to frustration, whereas insufficient challenge may result in boredom and reduced motivation (15). Time pressure may also negatively affect users with cognitive impairments (6). Although automated approaches can adapt difficulty according to performance (16), therapist-controlled adjustment remains a practical approach for tailoring activities to individual therapeutic requirements (17). Clinical judgement is particularly important because rehabilitation protocols need to account for therapeutic objectives, individual capabilities, fatigue, discomfort, and other factors that may affect participation (18). In rehabilitation involving children, these requirements are accompanied by the need to consider emotional and physical needs and interactions with caregivers (19, 20).

The collection and use of data introduce further design considerations. Privacy and data security are important in rehabilitation technologies, particularly when children are involved (21), and concerns about sensing technologies such as cameras and headsets may affect clinical acceptance (22). Evaluation approaches must also be appropriate for the intended users. Standardized usability instruments such as the System Usability Scale (SUS), Post-Study System Usability Questionnaire (PSSUQ), and Quebec User Evaluation of Satisfaction with Assistive Technology (QUEST) are commonly used to evaluate interactive health technologies (23), but their structure may not always be suitable for children or users with cognitive or communication difficulties. This highlights the importance of accessible and age-appropriate approaches to collecting user feedback.

Alongside subjective feedback, rehabilitation games can capture objective performance information. Commonly reported measures include task duration, session completion, gesture accuracy, and reaction time, although clinically relevant characteristics such as posture control, movement speed, harmony, and smoothness have been less frequently assessed (24, 25). Determining which measures are meaningful therefore requires alignment between technical capabilities and therapeutic requirements. More broadly, multidisciplinary collaboration among clinicians, engineers, and users is important for ensuring that rehabilitation technologies address therapeutic goals while remaining usable and appropriate for their intended population (26).

Despite these established principles, there remains limited practical guidance on how they should be translated into concrete design decisions during the development and clinical implementation of serious games for rehabilitation in children. Evidence suggests that locally developed games may benefit from greater consideration of user context, skills, and emotional needs (27), yet formalized approaches for structuring stakeholder involvement and translating clinical requirements into design decisions remain limited (28). Consequently, experience-based evidence from real clinical development settings is needed to complement existing high-level design principles.

This study addresses this gap through the iterative development of two serious games for upper-limb rehabilitation in children: Hungry Hamster, developed for use with a finger ladder, and Treasure Hunter, developed for use with a shoulder wheel. The systems were developed in collaboration with clinical stakeholders within a children's rehabilitation setting, with stakeholder feedback informing successive prototype modifications. The study presents the resulting clinician-informed design considerations together with descriptive usability findings from child participants. These considerations are subsequently compared with relevant literature to identify areas of alignment with existing principles, extensions arising within the present clinical context, and additional considerations identified through the development process.

## 2 Methodology

### 2.1 Study design

This study followed an iterative, user-centered design process to develop and refine two gamified rehabilitation tools, the finger ladder and the shoulder wheel, through the developed games Hungry Hamster and Treasure Hunter, respectively. Conducted at a children's rehabilitation center in Mexico, the study focused on integrating therapeutic objectives with engaging game mechanics tailored to the cognitive and physical capabilities of children.

### 2.2 Study context & participants

The study was conducted in collaboration with a multidisciplinary clinical team at the Center for Child Rehabilitation and Inclusion (CRIT) in Morelia, Mexico. CRIT serves a wide range of patients with neuromuscular and musculoskeletal conditions, providing a realistic clinical environment for observing usability and therapeutic alignment. A total of five distinct staff members participated in the focus group discussions: three were clinical participants (two rehabilitation physicians and one physical therapist), one was an informatics engineer responsible for integrating participant data into the center's databases, and one was the head of maintenance, who assisted with hardware installation, including adjusting the tablet height and addressing electrical considerations. Participation across the focus groups was role-dependent. The two rehabilitation physicians and the physical therapist attended all three focus groups, providing continuous clinical input throughout the iterative development process. The informatics engineer participated in Focus Groups 2 and 3, when data storage and database integration were addressed, while the head of maintenance participated in Focus Group 3, when installation and safety considerations were assessed. The two rehabilitation physicians, physical therapist, and head of maintenance also participated in the subsequent training session. Staff participants were selected using purposive sampling based on their direct involvement in upper-limb rehabilitation for children and in the deployment or maintenance of the rehabilitation devices. Inclusion criteria comprised clinical or technical personnel who were in therapy sessions involving the shoulder wheel and finger ladder. No formal exclusion criteria were beyond the requirement for direct involvement in the clinical workflow, ensuring that all participants could provide context-specific feedback on therapeutic relevance, usability, and system integration.

In total, 14 distinct children were involved in prototype testing: five engaged with Hungry Hamster (finger ladder) and eleven with Treasure Hunter (shoulder wheel). Two participants used both systems, as determined appropriate by their treating therapist based on clinical needs. In accordance with CRIT's child-safety and privacy requirements, the research team was not provided access to participant-level clinical records or detailed demographic and diagnostic information, including age, specific diagnosis, motor limitations, intellectual disability status, or functional classifications such as GMFCS and MACS.

Participating children were recruited using convenience sampling from children receiving upper-limb rehabilitation at the center. Inclusion criteria comprised children for whom the shoulder wheel or finger ladder exercises were part of their prescribed therapy and who were considered by their treating therapist to be able to interact with the game interface. No additional exclusion criteria were applied beyond standard clinical contraindications for the prescribed exercises. Selection and assignment to each game were determined exclusively by the treating therapists according to the child's prescribed rehabilitation programme, therapeutic goals, and functional abilities. Ethical approval for the study was granted by the institutional ethics committee, and informed consent was obtained from all participating staff and participating children or their legal guardians.

### 2.3 Design & development process

The development of the games was guided by an iterative, user-centered methodology involving close collaboration with clinical stakeholders at every stage. The primary goal was to create serious games that mirror therapeutic exercises while supporting motivation and engagement among children. Game design prioritized simplicity, inclusivity, and therapeutic relevance. The games were developed around two rehabilitation devices already used in routine children's therapy at CRIT: the finger ladder and the shoulder wheel. Within this clinical setting, these devices are used as general-purpose exercise tools to support upper-limb motor control, range of motion, and movement coordination. Their use with individual children is determined by the treating therapist according to the child's therapeutic goals and functional abilities.

The finger ladder and shoulder wheel were selected for gamification because clinicians identified the conventional exercises performed with these devices as repetitive and potentially insufficiently engaging. This work therefore focused on retrofitting the existing devices with interactive game elements. The gamified activities were also designed to incorporate coordination demands by requiring children to follow sequential targets on the finger ladder and movement patterns on the shoulder wheel.

Hungry Hamster gamifies the finger ladder. The ladder was fabricated using a Computer Numerical Control (CNC) machine and instrumented with capacitive sensors connected to an ESP32 microcontroller. In the initial prototype, eight of the ESP32's available capacitive input pins were used to detect interaction with eight of the 32 ladder rungs. Each capacitive input was individually calibrated to establish the threshold used to distinguish a touch from a non-touch condition. During gameplay, progression was registered only when instrumented rungs were detected sequentially in ascending order. The ESP32 prevented progression when instrumented rungs were skipped, thereby preventing the player from advancing the game by bypassing the expected movement sequence. The ESP32 maintained communication with the tablet throughout the session and transmitted the identifier of each detected rung together with its detection time. At the end of each session, these data were stored in a file and used to derive session-level measures including the time between adjacent detected rungs, total repetition time, and maximum height reached.

As the patient progresses up the ladder, detected movements were mapped to the game, causing the hamster on screen to climb a virtual tree and collect fruits until it reached its treehouse. The therapist-facing interface enabled session parameters to be configured and allowed completed and incomplete repetitions to be monitored. The complete game setup, including the electronic components, is shown in Figure 1.

**Figure 1:**
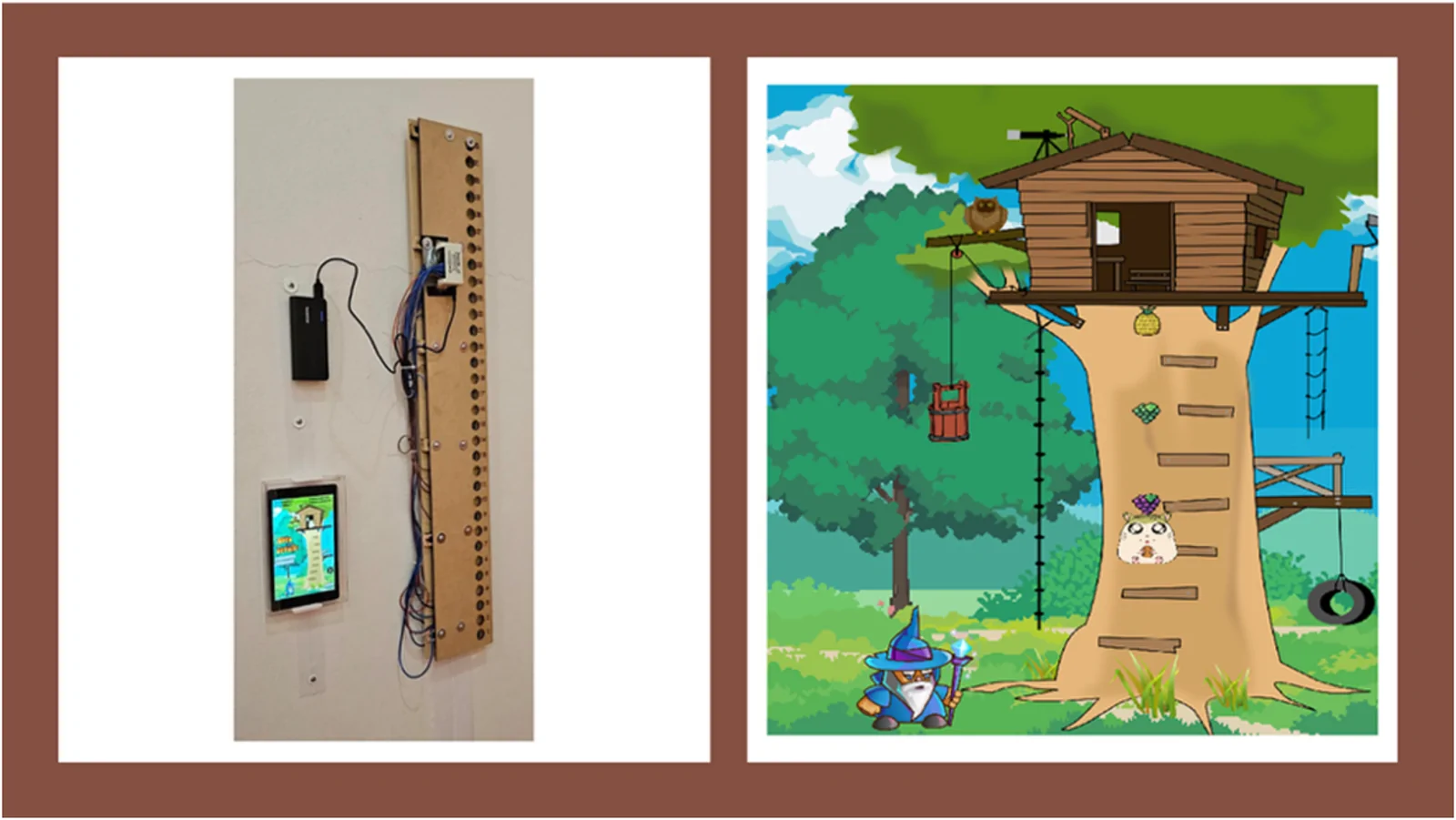
Hungry Hamster serious game for finger-ladder-based upper-limb rehabilitation in children, showing the gameplay environment, instrumented finger ladder, electronic components, and installed system at the rehabilitation center.

Treasure Hunter, based on the shoulder wheel, tasks the player with guiding a ship to collect treasures by aligning two on-screen balls: one representing a moving target and the other linked to the patient's actual movement. The patient-controlled ball is driven by data from a gyroscope-equipped shoulder wheel, with angular position transmitted to the tablet via Bluetooth. Successful alignment of the two balls results in the collection of virtual treasures. Shoulder-wheel orientation was measured using an MPU6050 inertial measurement unit (IMU) connected to an ESP32 microcontroller via the I^2^C protocol. Sensor data were sampled at 600 Hz and processed using an Arduino library implementing a complementary filter that fused accelerometer and gyroscope measurements to reduce gyroscope drift and accelerometer noise. For calibration, the sensor orientation was referenced to the zero-angle position of the shoulder wheel used by the game. The physical mounting orientation of the sensor was taken into account when establishing this reference; for example, positioning the sensor horizontally at the lower part of the wheel would correspond to an angular offset of −90° relative to the game's zero position. This reference was used to map subsequent wheel movements to the corresponding angular position within the game. The processed inclination angle was then transmitted via Bluetooth to the tablet and mapped to the patient-controlled ball in real time. Therapists could configure the required movement and game parameters according to individual therapeutic needs and functional abilities. The shoulder wheel hardware and in-game interface are shown in Figure 2.

**Figure 2:**
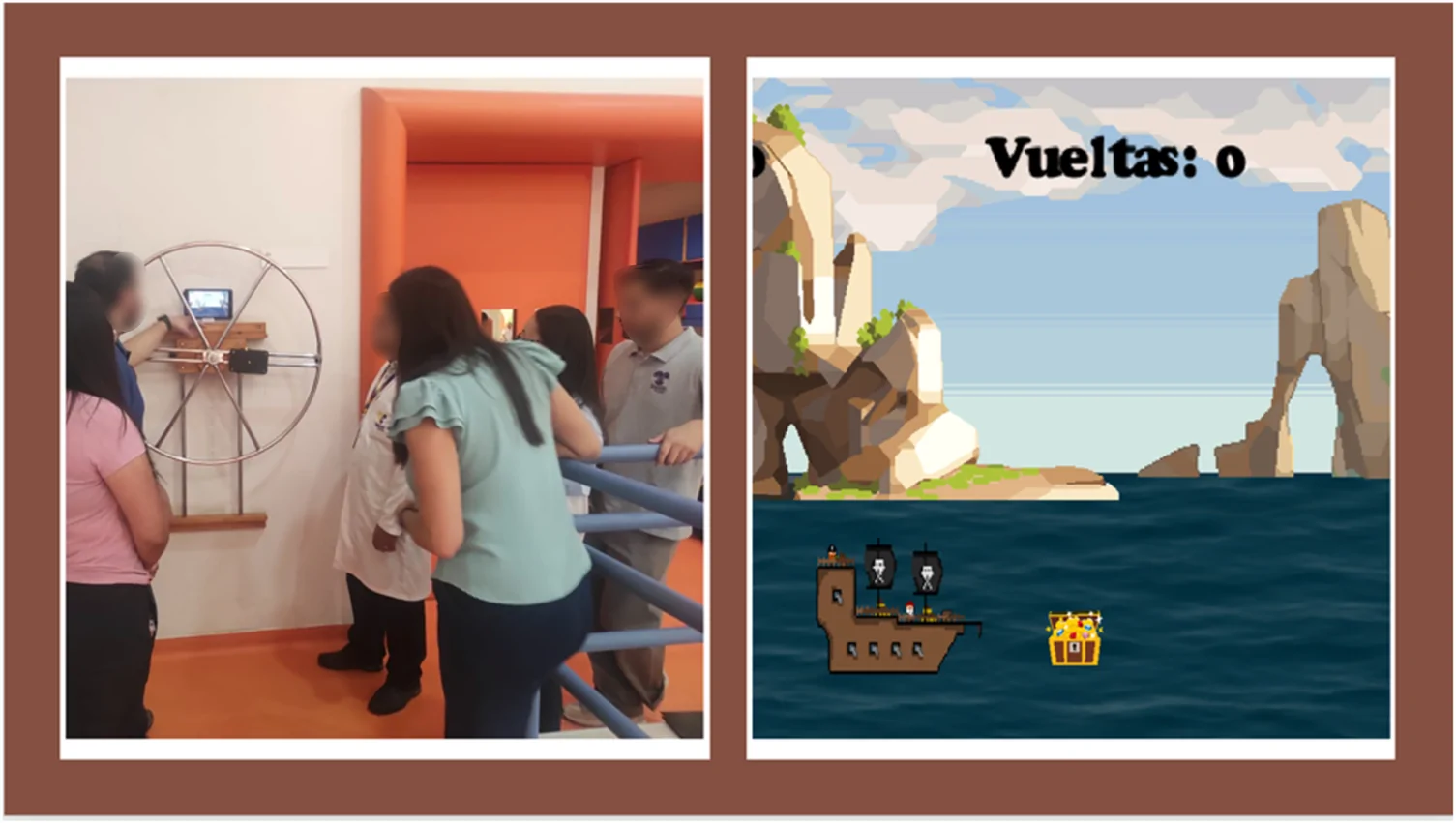
Treasure Hunter serious game for shoulder-wheel-based upper-limb rehabilitation in children, showing the gameplay environment, instrumented shoulder wheel, and installed system at the rehabilitation center. Reprinted with permission from Gamified Shoulder Wheel: Enhancing Pediatric Engagement and Data Collection by Infante-Sánchez, D., Bourazeri, A., Lira, T. C., Vázquez, I. L., Espino, E. E., Carrillo, P. E. R., and Alvarado, A. J. H. (2026), licensed under CC BY-NC-ND 4.0, International Journal of Serious Games, 13(2), 115–134.

Prototypes were developed to support upper-limb rehabilitation in children and included mechanical components designed to promote therapeutic movement. Devices were height-adjustable to suit individual patient needs. Games were implemented using the Android Game Development Kit (AGDK) and configured to run on tablets, featuring user-friendly interfaces for both children and clinicians. A clinician-facing configuration screen enabled adjustment of session parameters such as movement range, duration, and difficulty. Therapy sessions were automatically logged and stored locally, capturing metrics including session completion, timing between movements, range of motion, and number of repetitions. These data were later used to evaluate both gameplay performance and therapeutic alignment.

### 2.4 Focus groups & iterative feedback

Three structured focus group sessions were conducted for each game during the development process to gather iterative feedback. The sessions occurred at distinct stages of prototype development: Focus Group 1 was conducted after the initial game concepts and prototypes had been developed, Focus Group 2 after implementation of the first functional prototypes, and Focus Group 3 after the refined prototypes had been installed in the clinical setting. The intervals between sessions were not fixed. Following each focus group, the research team implemented the agreed modifications, and the subsequent session was scheduled once these changes had been completed. Participation in each session was determined by the expertise required at the corresponding stage of development.

Sessions were moderated by a member of the research team with experience in rehabilitation technologies and human–computer interaction, and had a duration of approximately 45–60 min. A semi-structured discussion format was used to ensure consistency while allowing participants to elaborate on clinically relevant issues. Core topics included therapeutic alignment of the proposed game mechanics, clinician-controlled configuration of movement parameters (e.g., angles, repetitions, and timing), usability of the hardware setup, pacing and difficulty adjustment according to individual patient needs, feedback modalities, and requirements for therapy-relevant performance data capture. These aspects informed prototype modifications but were not treated as independent outcome variables in the subsequent children's usability evaluation. All sessions were audio recorded, and the analytic approach consisted of a structured review of the transcripts to identify clinically relevant design requirements, usability issues, and configuration needs related to therapeutic implementation. Formal themes were not generated, and no coding framework or thematic analysis was applied, as the focus groups were used to guide iterative prototype development rather than to generate theory. During each session, proposed requirements and design modifications were discussed collectively by the participating clinical and technical staff, and agreement was reached on whether and how they should be implemented. The research team documented these agreed changes and incorporated them into the subsequent prototype iteration. The implemented modifications were then presented and assessed during the following focus group, allowing further requirements or refinements to be identified and agreed upon. Where differences in opinion arose, clinical decisions were guided by the treating therapists’ judgment regarding therapeutic requirements. To reduce the influence of individual researcher interpretation, design decisions were based on changes agreed collectively during the focus-group discussions rather than on themes independently inferred by the research team, and the resulting modifications were subsequently presented to the clinical participants for further assessment during the next development stage.

### 2.5 Data collection & clinical integration

Feedback was collected qualitatively through focus group transcripts, developer-clinician interactions, and observational notes gathered during prototype testing. Usability and therapeutic suitability were judged through consensus among the clinical team. Following completion of the three focus groups, the prototypes were finalized and installed in the clinical setting, and clinical staff completed the training session. Children gameplay testing was conducted only after this process had been completed; therefore, the children did not participate in the iterative design process. During these subsequent gameplay sessions, quantitative usability data were collected using a simplified, child-friendly questionnaire developed by the research team specifically for this exploratory study, as no previously validated standardized usability instrument (e.g., SUS or PSSUQ) was considered appropriate to accommodate the cognitive and developmental characteristics of the child population. It comprised 11 questions addressing children's self-reported enjoyment, understanding of the game mechanics, ease of use, engagement, motivation, emotional response, willingness to play again, and perceptions of performing the rehabilitation exercise with and without the game. Clinical input informed the questionnaire design to support age-appropriate comprehension. Specifically, the main clinical collaborator recommended using facial icons to represent children's degree of agreement, reflecting the approach commonly used by clinicians to communicate with children at the rehabilitation center. The collaborator also recommended limiting the response scale to five points to maintain simplicity. Accordingly, the questionnaire used a five-point Likert scale with facial-icon response options. Responses were coded as 1 = “Definitely not”, 2 = “No”, 3 = “Maybe”, 4 = “Yes”, and 5 = “Yes, definitely!” with higher scores representing more positive responses (Figure 3). Individual items were summarized descriptively, and no composite questionnaire score was calculated. The questionnaire was administered in game-specific versions, with selected items adapted to reflect the context of Hungry Hamster or Treasure Hunter. For clarity, a merged version is presented below to illustrate the shared structure across both implementations. While some items assess general enjoyment (e.g., willingness to play again), others specifically evaluate acceptance of the game as part of a rehabilitation routine.

**Figure 3:**
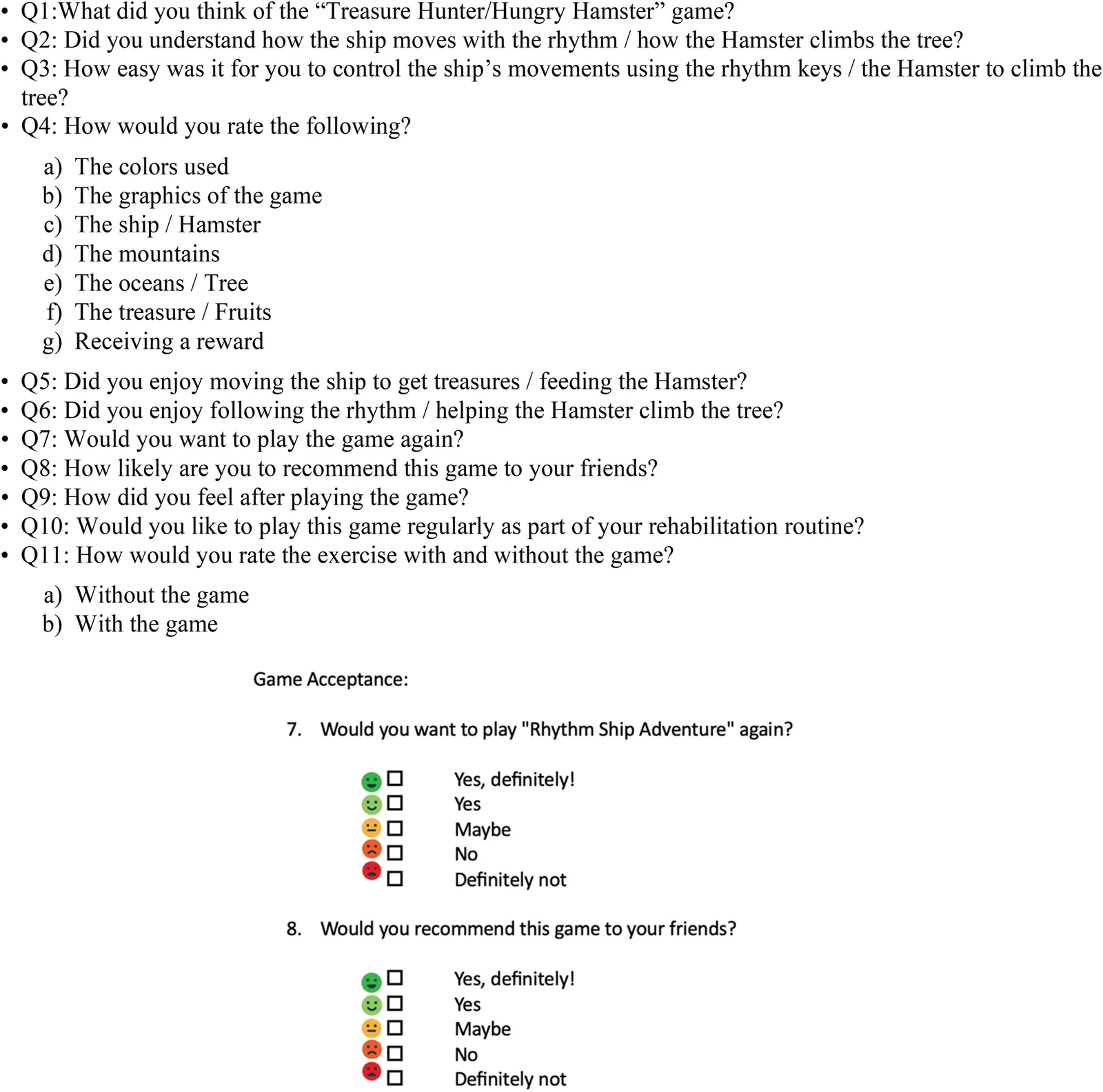
Child-friendly usability questionnaire used to evaluate Hungry Hamster and Treasure Hunter following gameplay, including items assessing enjoyment, understanding, ease of interaction, game features, motivation, emotional response, willingness to replay and recommend the game, and perceptions of rehabilitation exercise with and without the game. Responses were recorded using a five-point Likert scale with facial-icon response options.

The questionnaire was administered in Spanish, the native language of the participating children, immediately after each gameplay session with the assistance of a therapist or researcher when needed to ensure comprehension. Children responded using the emoji-based response options by selecting the option that best reflected their experience. No time limit was imposed, and clarifications were provided using standardized wording when necessary. Responses were recorded directly by the research team. Questionnaire content and responses reported in the manuscript were subsequently translated into English by a bilingual member of the research team with clinical knowledge of the terminology. No formal forward-backward translation or independent verification procedure was performed. Participants had prior experience performing the corresponding rehabilitation exercises without the game as part of their standard therapy, allowing Q11 to capture a direct comparison between traditional and gamified exercise. Objective performance data were stored in comma-separated values (CSV) files and included session summaries, movement metrics, and configuration parameters. These data were made available to therapists to support the monitoring of trends across sessions and potential adaption of therapy plans. Figure 3 presents a representative version of the questionnaire structure.

## 3 Results

### 3.1 Overview of findings

This section presents the findings from the iterative development and children prototype testing of Hungry Hamster and Treasure Hunter. The results are organized into two parts: the stakeholder-informed modifications arising from the iterative development process and the descriptive outcomes of the child-friendly usability questionnaire.

### 3.2 Iterative development findings

The iterative development process identified several requirements for adapting the two systems to the clinical setting. Stakeholder feedback primarily concerned accommodating individual movement capabilities, improving interaction with the rehabilitation devices, supporting safe clinical installation, and enabling the collection of therapy-relevant performance data. These requirements resulted in progressive modifications to both Hungry Hamster and Treasure Hunter across the three focus-group stages.

Following the initial prototype review, modifications focused on aligning movement tracking with therapeutic requirements, introducing clinician-facing configuration options, and accommodating children with different movement capabilities. For Hungry Hamster, the finger ladder was adapted to increase movement range and support sequential movement tracking, while Treasure Hunter was initially configured to support a greater range of shoulder-wheel movement. Following evaluation of the functional prototypes, sensor coverage and data capture were expanded, and configurable time- and repetition-based session parameters were introduced. The systems were also adapted to record therapy-relevant measures, including inter-step timing, movement timing, range of motion, repetitions, and session duration, as applicable to each device. During the final clinical installation stage, further modifications addressed physical installation and safety, data storage and compatibility, and the refinement of auditory and visual feedback. For example, Hungry Hamster was modified to use rechargeable battery power following a safety recommendation, while Treasure Hunter was adapted to support partial angular movements rather than requiring complete shoulder-wheel rotations to accommodate children with restricted ranges of movement. An additional training session was subsequently conducted with clinicians to support implementation. Table 1 summarizes the stakeholder feedback and corresponding modifications implemented at each stage of development.

**Table 1:** Stakeholder feedback and corresponding modifications to Hungry Hamster and Treasure Hunter across the iterative development stages, including participants involved, identified requirements, and changes implemented for the finger-ladder and shoulder-wheel systems.

Development stage	Participants involved	Main feedback/requirements	Hungry Hamster (Finger Ladder): changes implemented	Treasure Hunter (Shoulder Wheel): changes implemented
Initial concept and low-fidelity prototype	__	Initial concepts prepared for clinical review	Hamster climbs a virtual tree through activation of capacitive sensors on ladder rungs and collects fruit	Player guides a ship to collect treasures by aligning a target with movement captured from the gyroscope-equipped wheel
Focus Group 1: Initial prototype review	2 rehabilitation physicians+1 physical therapist (*n* = 3)	Align movement tracking with therapeutic goals; provide therapy configuration; accommodate limited mobility and slower responses	Finger ladder size increased; clinician-facing configuration added; gameplay designed without externally imposed pacing	Movement range extended to full rotations; clinician-facing configuration added; pacing adjusted to accommodate slower movements
Focus Group 2: Functional prototype review	2 rehabilitation physicians+1 physical therapist+1 informatics engineer (*n* = 4)	Improve sensor coverage and data capture; provide flexible goal setting; ensure compatibility with clinical data systems	Sensor coverage expanded; inter-step time, repetitions, and session duration logged; time- and repetition-based configurations introduced; CSV storage adopted	Sensor coverage expanded; range of motion, movement timing, repetitions, and session duration logged; time- and repetition-based configurations introduced; CSV storage adopted
Focus Group 3: Clinical installation and final evaluation	2 rehabilitation physicians+1 physical therapist+1 informatics engineer+1 head of maintenance (*n* = 5)	Evaluate final functionality; verify data compatibility and installation; refine feedback and rewards	Data capture and CSV compatibility confirmed; installation reviewed; auditory cues and visual rewards refined	Data capture and CSV compatibility confirmed; installation reviewed; auditory cues and visual rewards refined
Training and children testing	2 rehabilitation physicians+1 physical therapist+1 head of maintenance (*n* = 4) 14 children	Support clinical implementation and subsequent prototype testing	Clinician training completed; 5 participating children subsequently tested Hungry Hamster	Clinician training completed; 11 participating children subsequently tested Treasure Hunter

### 3.3 Usability findings from child participants

Given the exploratory nature of the study, the small sample sizes, and the ordinal five-point response scale, the child-friendly questionnaire findings were analyzed descriptively without inferential statistical testing. Questionnaire data were available for five participants who evaluated Hungry Hamster and eleven participants who evaluated Treasure Hunter. Item-level means and standard deviations are presented in Figures 4, 5, while medians, ranges, and response distributions are reported below to provide additional information on the distribution of responses. For Hungry Hamster, responses across Q1–Q10 were generally toward the positive end of the response scale, with item-level means ranging from 3.8 to 4.6. For Q11, the exercise without the game had a mean rating of 2.6 (median = 3, range = 1–4), whereas the exercise with the game had a mean rating of 4.4 (median = 5, range = 3–5). Three of the five participants rated the exercise with the game as 5, one as 4, and one as 3. For Treasure Hunter, item-level means across Q1–Q10 ranged from 3.75 to 4.25. For Q11, the exercise without the game had a mean rating of 2.875 (median = 3, range = 1–4), whereas the exercise with the game had a mean rating of 4.25 (median = 4, range = 4–5). Nine of the eleven participants rated the exercise with the game as 4 and two as 5. Overall, the descriptive questionnaire findings indicate more positive ratings for performing the rehabilitation exercises with the games than without them.

**Figure 4:**
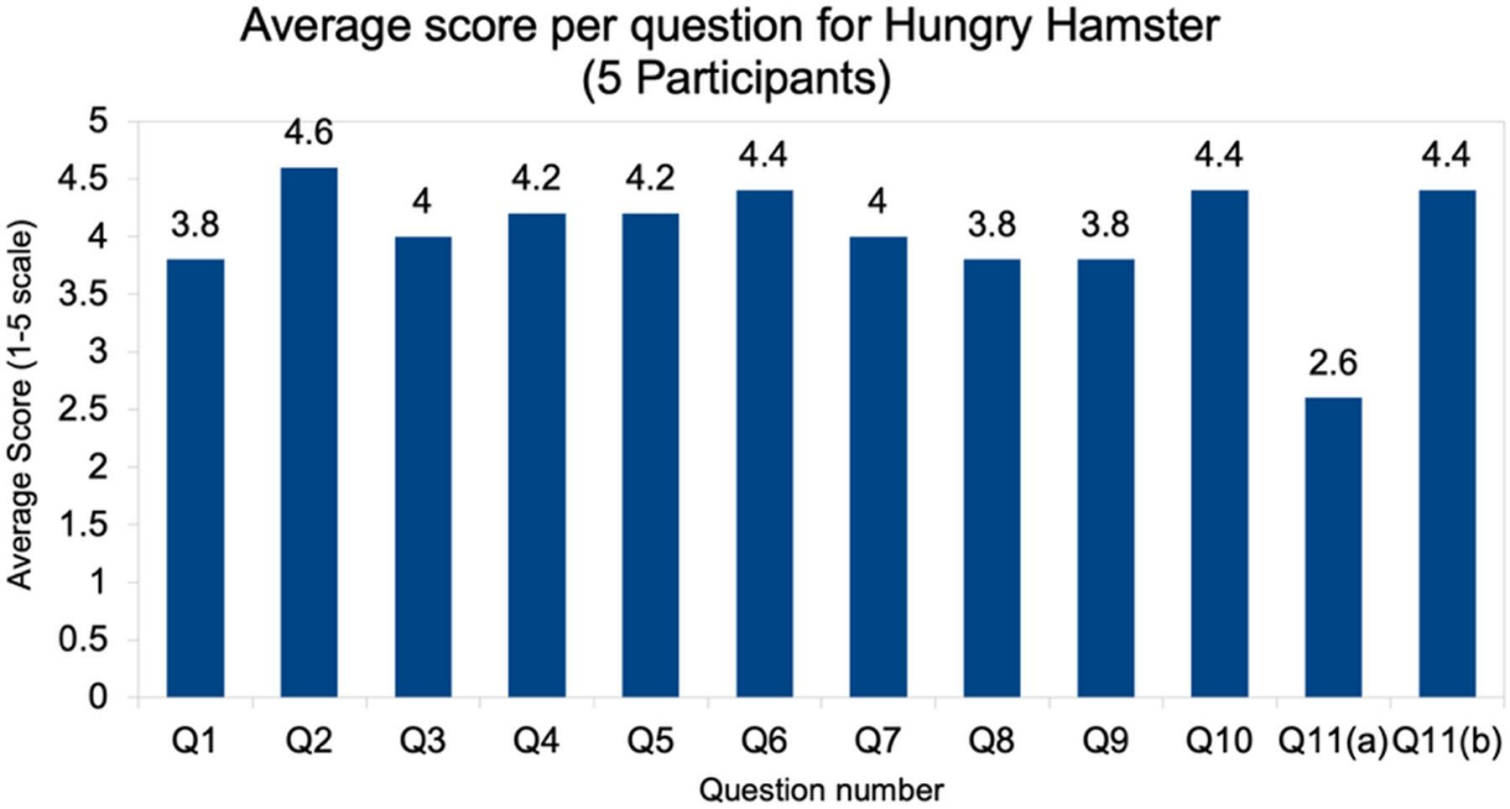
Descriptive usability ratings for Hungry Hamster reported by five participating children following gameplay. Bars represent mean item-level scores on the five-point response scale, with higher scores indicating more positive responses.

**Figure 5:**
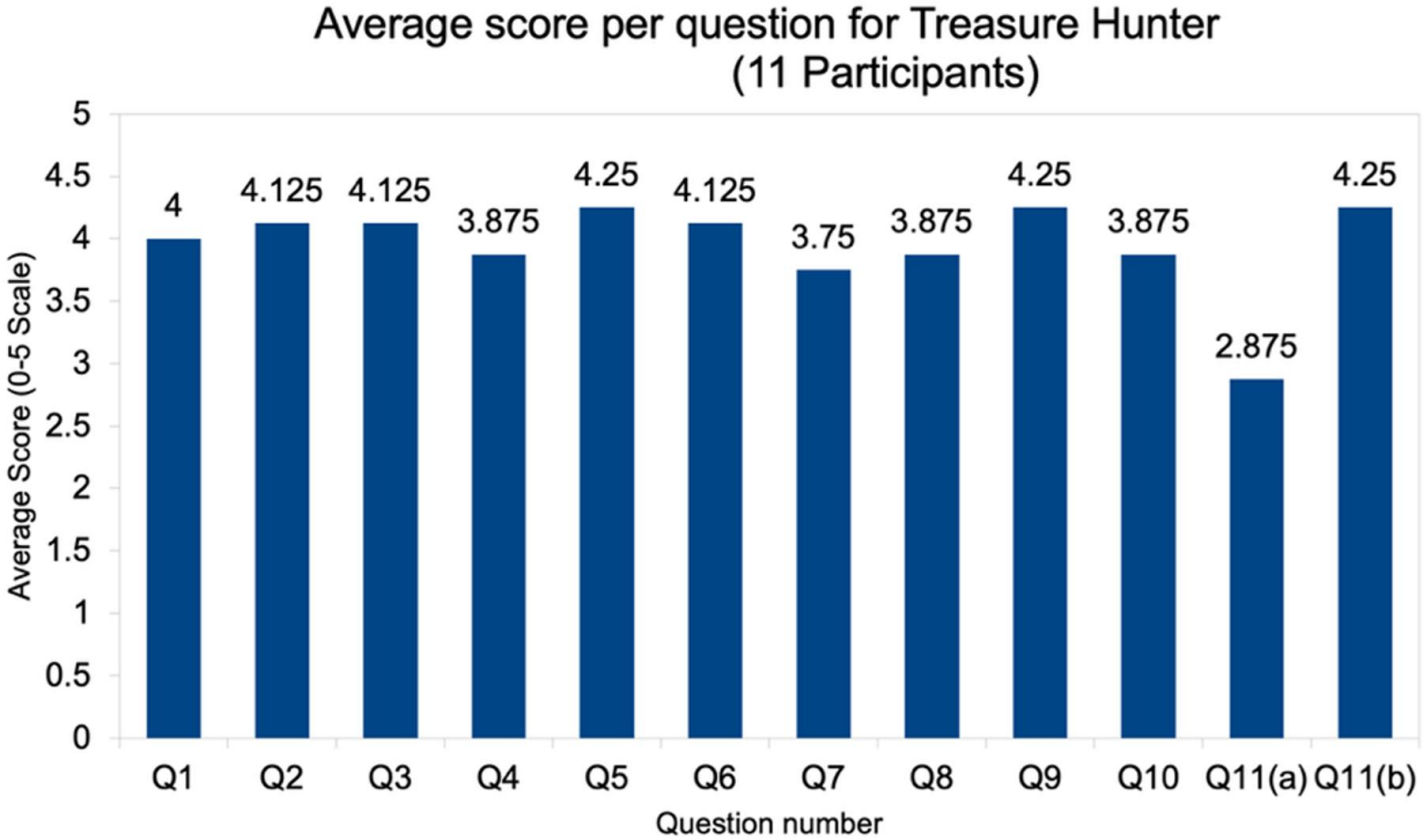
Descriptive usability ratings for Treasure Hunter reported by eleven participating children following gameplay. Bars represent mean item-level scores on the five-point response scale, with higher scores indicating more positive responses.

## 4 Discussion

This section presents and discusses preliminary clinician-informed design considerations derived from the collaborative development of Hungry Hamster and Treasure Hunter, and contextualized through relevant findings from the literature. The literature review was narrative rather than systematic and was used to contextualize the clinician-informed observations within relevant existing research. Table 2 compares design principles identified in the literature with considerations arising from the iterative development of the two systems. The table distinguishes considerations that were consistent with existing principles, those that extended them within the present clinical context, and additional considerations identified during the development process. Furthermore, not all aspects discussed were evaluated as study outcomes. For example, difficulty was configured by clinicians, while privacy and security informed implementation decisions, but neither was formally assessed as an independent outcome variable. Feature extraction and data capture were implemented for therapy-relevant metrics, but the resulting measures were not formally validated as clinical outcome measures.

**Table 2:** Comparison of literature-derived design principles with clinician-informed design considerations identified during the iterative development of Hungry Hamster and Treasure Hunter for upper-limb rehabilitation in children, including their relationship to existing literature.

Design aspect	Literature-derived design principle	Clinician-informed consideration from this study	Relationship to literature
Therapeutic gesture integration	Therapeutic gestures should be integrated into the virtual environment (11).	Game actions should closely mirror the physical rehabilitation exercise; for example, the hamster progresses as the child moves along the finger ladder, while movement of the shoulder wheel controls the Treasure Hunter game.	Consistent with literature — reinforces correspondence between therapeutic movement and game action.
Calibration and configuration	Game mechanics should be adaptable to users’ motor capabilities through calibration (11).	Clinician-facing configuration should allow therapeutic parameters such as angles, repetitions, and timing to be adjusted before each session.	Extends the literature in this clinical context — emphasizes clinician-controlled configuration according to therapeutic requirements.
Performance monitoring	Player performance should be monitored and recorded for evaluation (11).	Objective data capture should focus on therapy-relevant measures applicable to each system, including movement timing, range of motion, session duration, and repetition count.	Extends the literature in this clinical context — clinicians and technical stakeholders specified measures relevant to monitoring rehabilitation activity.
Difficulty and session configuration	Challenge should be progressively adapted to users’ abilities and performance (11, 16).	Difficulty should be configurable by the therapist through parameters such as movement requirements, repetitions, and timing, while also considering fatigue, frustration, engagement, and the child's response to the activity.	Extends the literature in this clinical context — highlights clinician judgement and individual responses alongside performance. Difficulty was configured but not evaluated as an independent outcome.
Content diversity	Content diversity can support individual preferences and continued engagement (11).	Optional variations in visual content may help reduce monotony, provided that they do not interfere with the therapeutic movement or purpose of the exercise.	Consistent with literature, with a clinical caveat — variation should not compromise therapeutic integrity.
Feedback and rewards	Positive reinforcement and multimodal feedback can support engagement (9, 11).	Visual and auditory rewards should provide clear positive feedback for successful movements while avoiding feedback that could unnecessarily increase frustration.	Extends the literature in this clinical context — emphasizes frequent positive reinforcement and avoidance of discouraging failure mechanics.
Narrative simplicity	Simple gameplay and avoidance of losing, dying, or failure mechanics may be appropriate in rehabilitation games; narrative and character development can support engagement (7, 8).	Simple, goal-oriented narratives should provide context for the therapeutic activity without introducing unnecessary cognitive demands or failure mechanics.	Consistent with literature — the two prototypes operationalized these principles through simple rehabilitation-oriented game objectives.
Visual design	Simple backgrounds and avoidance of excessive visual stimulation are recommended (10, 12).	Decorative elements should provide visual interest without distracting from the therapeutic task; movement of non-essential elements should remain slow and non-interfering.	Extends the literature in this clinical context — provides practical considerations concerning the movement of decorative elements.
Inclusivity and accessibility	Game content should account for the patient's condition, age, preferences, knowledge, and motivation (12, 14).	Visual and auditory feedback, gameplay pacing, and the physical setup should be configurable to accommodate different functional and interaction needs; adjustable equipment can also support wheelchair users and children of different heights.	Extends the literature in this clinical context — identifies practical software and physical-accessibility considerations arising during implementation.
Individual differences in engagement	Personal characteristics and intrinsic motivation should be considered in rehabilitation game design (14).	Game configuration should account for individual differences in responses to competition, frustration, fatigue, and engagement, including the need for rest periods or shorter sessions where appropriate.	Additional children-specific consideration identified — highlights the role of clinician judgement when adapting gameplay to individual responses.
Privacy and security	Privacy and data security are particularly important when designing technologies for children; camera- and headset-based systems may introduce additional concerns (21, 22).	Privacy was considered through implementation choices including local data storage and avoidance of camera- or microphone-based interaction.	Consistent with literature — privacy informed implementation decisions but was not formally evaluated as a study outcome.
Feature extraction and data capture	Duration, task completion, gesture accuracy, and reaction time are commonly captured, whereas measures such as posture control and movement smoothness are less frequently assessed (24, 25).	Therapy-relevant metrics applicable to the respective systems, including movement timing, range of motion, session duration, repetition count, and arm usage, should be captured to support clinical monitoring.	Extends the literature in this clinical context — the development process identified clinically relevant data requirements; broader movement-quality measures were not quantitatively assessed.
Therapy time and repetition count	Therapy duration and repetition count are not strongly emphasized as distinct design requirements in the reviewed literature.	Recording therapy duration and repetition count was identified during the development of both systems as important for clinical monitoring.	Additional clinical consideration identified — highlights simple measures considered useful by the participating clinicians.
Children usability assessment	Standard usability instruments may not always be appropriate for children with differing cognitive and developmental needs (23).	Child-accessible assessment approaches, including simplified wording and visual response scales, should be considered when evaluating therapeutic games.	Additional methodological consideration identified — the present study used a child-friendly facial-icon questionnaire, but the instrument was not validated and requires validation in future work.

### 4.1 Privacy & data security

Privacy and data security are critical considerations in the design and implementation of serious games (21), particularly when sensitive patient information is involved (29). The risk of data breaches, which could lead to the unauthorized disclosure of personal health information, poses a significant challenge. This concern is further amplified when systems incorporate cameras or microphones to capture images, audio, or environmental data related to the patient. A particularly pressing issue arises when data is collected without the system being actively in use, raising ethical and legal questions about consent and data management. These concerns become even more pronounced when the patients are children, as they represent a vulnerable population requiring heightened protections. Consequently, it is imperative for system development engineering teams to prioritize robust security measures to ensure that all data collected is securely stored, managed, and protected against unauthorized access or misuse.

### 4.2 Game story

Commercial games often incorporate narrative elements to enhance player engagement and enjoyment. Similarly, serious games for therapeutic use can benefit from storytelling that fosters children's involvement in treatment activities. In the development of Hungry Hamster and Treasure Hunter, care was taken to avoid concepts such as failure or dying (8). Instead, the games emphasized continuous rewards to maintain motivation.

In Hungry Hamster, a clinical collaborator emphasized the effectiveness of a simple narrative; placing fruits on the rungs of a virtual tree to feed a hungry hamster. Framing the activity as “*let's go feed the hamster because it is skinny*” made the task more appealing and gave children a meaningful objective beyond the repetition of motor tasks like moving up the ladder or counting steps. Similarly, Treasure Hunter featured a straightforward story in which players navigated the sea to collect treasures. The game avoided failure mechanics and used simple, intuitive rules to make goals easily understandable for children (7). These examples underscore the value of integrating clear and compelling narratives into serious games. Unlike commercial titles that may rely on complex storylines, therapeutic games should prioritize simplicity and accessibility to engage children across a range of ages and cognitive abilities.

### 4.3 Graphical elements

The developed games avoided visual clutter that could distract participants from the primary therapeutic activity (10). However, decorative elements and sounds were included to enhance patient engagement. For instance, in the game Treasure Hunter, decorative elements such as mountains and clouds were incorporated to enhance visual appeal. A clinician involved in the evaluation noted that the slow movement of the clouds was particularly effective, as it added dynamism without causing distraction for the children. Overall, feedback regarding the quantity and speed of decorative elements in the game was positive, highlighting the importance of balancing visual engagement with functional clarity. While additional graphical elements could be introduced to align the aesthetic quality of serious games more closely with that of commercial games, care must be taken to ensure that such enhancements do not overwhelm or distract children. In Hungry Hamster, a wizard appears at the start of the game to cast a spell, adding initial dynamism without disrupting gameplay. After this introduction, the wizard does not interfere, allowing the player to focus on controlling the hamster's ladder-climbing activity. This careful balance between aesthetic enhancement and therapeutic clarity underscores the importance of purposeful visual design in rehabilitation games for children.

### 4.4 Constant rewards

Many of the serious games reviewed in the literature are task-oriented, with the primary objective of expanding the range of movement by guiding users from one point to another. Success is typically measured by reaching a goal, which increases the player's score. However, incorporating constant rewards has been shown to enhance user engagement, reduce frustration, and provide immediate feedback, confirming that the movement performed is correct (11). For example, in the game Hungry Hamster, the primary objective is to guide the hamster to a treehouse. Along the way, fruits placed on the rungs of the treehouse serve as rewards; when collected, they trigger auditory feedback, such as chewing sounds, and visually enlarge the hamster. Additionally, rungs without fruits produce a stepping sound, offering auditory cues that reinforce the correct sequential movement which can be beneficial for players with learning disabilities (6). If the patient attempts to skip rungs to reach the treehouse more quickly, the character remains stationary, signaling that the correct sequence was not followed. This design not only enhances engagement but also provides real-time feedback on the accuracy of the movement, thereby supporting both motivational and therapeutic goals.

### 4.5 Inclusivity

Inclusivity emerged as an implicit yet central theme in discussions surrounding the gamification of rehabilitation devices. Serious games for children's therapy must account for both the cognitive abilities (7) and physical needs (20) of users. CRIT, a therapeutic center specializing in children's care, serves children with a wide range of conditions, including autism, Down syndrome, cerebral palsy, and various physical disabilities. To address this diversity, several strategies were proposed to ensure that gamified rehabilitation tools remained accessible, engaging, and meaningful for all participants.

Design recommendations included the use of simple, narrative-driven games such as a scenario in Hungry Hamster where a hamster collects fruit to reach its home which were intentionally easy to understand. Other accessibility-focused features included cheerful and louder auditory feedback, a reduction of distracting visual elements, and slower-paced gameplay, all aimed at supporting children with cognitive or sensory processing challenges. However, clinicians also reported age-related differences in engagement. While most children responded positively to the games, older participants (approximately 12 years and above) tended to find the narratives and visual style less stimulating, whereas younger children and those with cognitive impairments benefited from the simplified interactions and clear objectives. These observations suggest that serious games for children's rehabilitation should incorporate adaptive content tailored to the user's age and cognitive level, balancing simplicity with sufficient challenge to maintain motivation.

Physical accessibility was also a key focus. For example, height-adjustable tables secured with Velcro straps were suggested to accommodate children of different statures, including those using wheelchairs. These considerations reflect a commitment to creating rehabilitation tools that are not only therapeutically effective but also adaptable to diverse physical and cognitive profiles. These adjustments align with universal design principles, emphasizing flexibility, simplicity, and equitable use to ensure accessibility for all children, regardless of ability.

### 4.6 Difficulty

The implementation of automatic difficulty adjustment mechanisms has been proposed as a strategy to maintain player engagement and foster improvement in therapeutic exercises by progressively increasing goals, such as wider angles, greater heights, or additional repetitions. Simple algorithmic solutions, often based on historical performance data such as success or failure rates, have been suggested to dynamically modify these goals (16). However, there remains a notable gap in the literature regarding the consideration of emotional states, pain levels, and medication effects during exercise performance, all of which can significantly influence therapy outcomes (18). This oversight is particularly critical in children, as highlighted by clinical practitioners who have observed that children may exhibit varying levels of willingness to participate in therapeutic activities. Consequently, monitoring the emotional state of children during exercise and adapting therapy objectives accordingly has been emphasized as a crucial factor in optimizing therapeutic outcomes.

Clinical participants have further categorized children into two distinct groups based on their behavioral and emotional responses. The first group comprises children who exhibit a competitive disposition, for whom therapy goals can be enhanced by increasing speed, repetition counts, or range of motion. The second group consists of children who are prone to frustration, requiring a more flexible approach that minimizes time constraints, repetition targets, or rigid goal-setting. In such cases, the role of the clinician becomes paramount, as they must continuously assess the emotional and cognitive state of the child, which can be influenced by factors such as medication, stress, and other external variables. Based on the child's willingness and disposition, therapists are tasked with adjusting therapy goals to ensure both engagement and therapeutic efficacy. This adaptive approach highlights the importance of integrating emotional and cognitive assessments into therapeutic interventions for children. By observing patients during therapy (17), clinicians can adjust treatment parameters and goals in real time.

An additional consideration in neurorehabilitation for children is covert fatigue, in which children may demonstrate preserved cognitive abilities but reduced endurance and increased susceptibility to overstimulation (30). This phenomenon may be exacerbated in game-based therapy, as young children and those with mild cognitive impairments must simultaneously observe the physical exercise and map it to on-screen objectives, increasing cognitive load. Even when the motor task is within the child's physical capacity, this additional processing demand can reduce endurance and lead to disengagement. Prolonged gameplay or visually complex environments may therefore contribute to fatigue despite adequate task comprehension. Therapeutic games should support scheduled or adaptive rest periods, reduced visual and sensory load, and shorter, modular sessions that maintain therapeutic benefit while minimizing fatigue. These strategies can help sustain engagement and optimize therapeutic outcomes, particularly for children with brain injury and related conditions.

### 4.7 Options

A notable limitation of the developed games is the absence of optional or customizable features, which may contribute to patient disengagement over extended periods of use. Long-term repetition of the same game mechanics and visual elements can lead to monotony, potentially reducing motivation and adherence to the therapeutic regimen. While game development is inherently time-intensive, a feasible solution to mitigate this issue could involve incorporating selectable options, such as interchangeable backgrounds or characters. These modifications, though relatively simple to implement from a coding perspective, could create the perception of variety and novelty, thereby aligning more closely with the preferences and interests of children. However, it is important to note that radical alterations to the game design are not advisable, as the game must maintain a close correspondence with the therapeutic activity it is intended to mirror. The primary objective of ensuring that the gameplay accurately reflects the physical exercise remains paramount. Therefore, while minor aesthetic changes, such as variations in background landscapes or character designs, can enhance engagement, they should not compromise the therapeutic integrity or the alignment between the game and the intended rehabilitation goals. This approach was reinforced by a clinician, who noted: “*While the game is on an electronic device, children find it appealing because they’re already accustomed to playing with technology”.*

### 4.8 Mirroring activity

The incorporation of mirroring activities, where virtual or game-based elements closely replicate the physical exercises being performed, has been widely recommended in the literature. This approach aims to enhance patient engagement and ensure alignment between therapeutic objectives and gameplay. However, the use of commercial games in therapeutic settings has been cautioned against, as it may inadvertently shift focus away from therapeutic goals (21, 31, 32) and promote incorrect or inappropriate movements, potentially confusing patients about the intended physical activity. Clinicians have emphasized the importance of ensuring a close correspondence between the exercise and the game to maintain therapeutic integrity and effectiveness (10) which can be beneficial for patients with cognitive disabilities to better understand the exercise to be performed. Additionally, it was suggested by one clinical participant that the finger ladder could be decorated with visual elements that mirror those in the game by painting a tree on the physical ladder and drawing the fruits. This strategy is proposed to facilitate a clearer understanding of the connection between the game and the physical exercise, thereby reducing cognitive load and enhancing patient comprehension.

### 4.9 Feature extraction

Feature extraction is a critical consideration (24), as the clinical value of metrics can vary widely. Focusing on clear indicators of range of motion such as achieving target angles and measuring time between shoulder wheel movements, or tracking repetition count and duration in the finger ladder, enables automated, objective measurement. This facilitates performance evaluation across repetitions and sessions, allowing clinicians to monitor patient progress more effectively. Clinical participants emphasized that such metrics are often overlooked in standard practice, despite their potential value.

One clinician noted that traditional rehabilitation tools rely heavily on subjective observation, making accurate data recording difficult. For example, one therapist might describe a task as “fast” if completed in two minutes, while another might interpret “fast” as 30 s. Objective timing data thus offers a clearer basis for tracking patient improvement.

The literature further highlights the importance of capturing rhythm, synchronization, and movement trajectory to assess not only end-range targets but also motion quality during intermediate phases (25). The proposed approach includes time-stamping the full trajectory such as timing each step on the finger ladder or pacing control on the shoulder wheel, which was recognized as a means of identifying motor difficulties within specific movement ranges. Increased time in certain segments may indicate areas of limitation. Setting a target movement speed on the shoulder wheel was also seen as a useful strategy to support motor control. One medical professional emphasized the benefit of adding a target ball to guide motion, noting its therapeutic value in promoting smoother and more controlled execution, and stated: “*It's beneficial that it's a rhythmic movement because it's not just about moving the wheel quickly but also about trying to maintain control of the speed. For instance, we have patients with spastic hemiparesis where they don't have good control of half of their body, especially the hand more so than the foot. Cognitively, they’re fine and can understand instructions, but they have trouble controlling fine motor skills. We can easily improve gross motor skills, and this idea can greatly help to improve fine motor skills because it involves maintaining a steady pace, following the speed”.*

A well-designed feature extraction system for serious games should include methods to measure and track range of motion for intra-and inter-session evaluation. Additionally, monitoring trajectory and speed appears useful for analyzing movement between initial and final targets, with potential benefits for improving fine motor control and a way to “zoom” problematic ranges of movement.

The medical team stressed the importance of monitoring inter-step times for the finger ladder and analyzing them through averaged values across four distinct step groups. This method offers granular insights into movement fluidity while pinpointing specific problematic angles or ranges. Additionally, they emphasized the need for consistent performance tracking across therapy sessions, particularly given that multiple therapists might be involved in a patient's care.

### 4.10 Child-friendly usability questionnaires & assessments

In light of differences in user engagement and comprehension, it is important not only to assess overall satisfaction, but also to evaluate individual design components. Conventional usability assessments often evaluate systems as a whole, without isolating feedback on specific elements such as background visuals, audio design, or interactive features. The use of targeted items in this study enabled more detailed insights into which aspects of the games supported engagement and comprehension. Clinicians observed that while most children responded positively to the games, older children (approximately 12 years and above) tended to find the narratives, graphics, and mechanics less stimulating. Conversely, younger children and those with cognitive impairments benefited from simplified interactions and clear objectives, although some required additional guidance to understand certain game mechanics. These observations indicate that serious games for rehabilitation in children should incorporate adaptive content tailored to the user's age and cognitive level, balancing simplicity with sufficient challenge to maintain motivation. Young children and those with mild cognitive impairments may experience cognitive overload when required to simultaneously observe the physical exercise and map it to on-screen objectives, which can reduce engagement despite adequate motor ability. The limited availability of standardized child-friendly usability instruments in the current literature highlights the need for the development and validation of age-appropriate, low-cognitive-load evaluation tools for serious games in rehabilitation contexts.

### 4.11 Study limitations

This study has several limitations that should be considered when interpreting the findings. First, the sample size was small, particularly for the child-friendly usability questionnaire, which limits the generalizability of the results. As a pilot, exploratory study, the quantitative outcomes were analyzed using descriptive statistics only, and no inferential statistical testing was performed. Consequently, the observed differences in affective ratings between gamified and non-gamified exercises should be interpreted as indicative trends rather than definitive evidence of effectiveness. Second, detailed demographic and clinical information of the participating children including age, specific diagnoses, motor limitations, intellectual disability status, and standardized functional classifications such as GMFCS and MACS, were unavailable to the research team because of CRIT's child-safety and privacy requirements. Given the small sample, sufficiently detailed aggregated reporting could also pose a risk of participant identification. Consequently, the relationship between specific participant characteristics and usability outcomes could not be examined, limiting the extent to which the findings can be interpreted across different clinical and functional profiles in children.

Third, the study-specific child-friendly questionnaire, while designed to be developmentally appropriate, was not a previously validated instrument. Although clinical input informed its development to support age-appropriate comprehension, no formal content-validation or reliability assessment was conducted, and its psychometric properties (e.g., reliability and construct validity) were not formally evaluated. Furthermore, the questionnaire was administered in Spanish and subsequently translated into English for reporting by a bilingual member of the research team, without a formal forward-backward translation or independent verification procedure. Future work should focus on the development and validation of standardized, low-cognitive-load usability instruments for children. Fourth, focus group findings were derived from a small number of clinical and technical staff within a single rehabilitation center. While this facilitated context-specific insights and close collaboration, it may limit transferability to other clinical settings with different workflows, resources, or patient populations.

Finally, the study evaluated short-term usability and engagement during prototype testing rather than long-term adherence, functional outcomes, or comparative therapeutic effectiveness. Longitudinal studies with larger and more diverse samples are needed to assess sustained motivation, clinical impact, and integration into routine rehabilitation practice. Despite these limitations, the study provides preliminary evidence supporting the feasibility of gamifying conventional rehabilitation tools and offers design recommendations grounded in clinical collaboration.

## 5 Conclusion

This study contributes exploratory, clinician-informed insights into the development of serious games for children. Through an iterative process involving structured focus group sessions with medical professionals, we integrated domain-specific feedback into both the mechanical design and game mechanics. The approach emphasized inclusivity, therapeutic relevance, and usability, resulting in tools that address the diverse cognitive and physical needs of children. A key contribution of this work is the identification and implementation of clinically meaningful features for automatic data capture, such as range of motion, movement timing, and repetition tracking. These metrics, often overlooked in traditional rehabilitation practice, enable objective assessment of patient progress and reduce reliance on subjective observation. Furthermore, the study applied inclusive design principles such as adjustable hardware, simplified narratives, and adaptive sensory feedback to promote accessibility for children with varying physical and cognitive conditions. The feedback from clinical participants also highlighted the importance of customizable gameplay pacing and engagement strategies tailored to individual user profiles. By comparing clinician perspectives with current gamification literature, the study reveals critical gaps in existing frameworks, particularly regarding their applicability to children's rehabilitation. The findings contribute to bridging the divide between theoretical game design models and real-world clinical implementation, offering preliminary, clinician-informed design considerations for the future development of serious games in healthcare. Future work should focus on formalizing these clinician-informed design considerations into structured development guidelines, as well as evaluating the therapeutic efficacy of the proposed tools in larger, longitudinal studies. Additional research is needed to develop and validate standardized, child-friendly questionnaires for serious game evaluation and to explore multilingual interface adaptation to enhance linguistic accessibility across diverse clinical contexts.

## Data Availability

The raw data supporting the conclusions of this article will be made available by the authors, without undue reservation.
